# The comparative energetics of the turtles and crocodiles

**DOI:** 10.1002/ece3.8996

**Published:** 2022-06-11

**Authors:** Nina Marn, Sebastiaan A. L. M. Kooijman

**Affiliations:** ^1^ Division for Marine and Environmental Research Rudjer Boskovic Institute Zagreb Croatia; ^2^ School of Biological Sciences The University of Western Australia Crawley WA Australia; ^3^ Department of Theoretical Biology VU University Amsterdam Amsterdam The Netherlands

**Keywords:** add‐my‐pet collection, dynamic energy budgets, life history, metabolism, population growth rate, traits

## Abstract

The Add‐my‐Pet collection of data on energetics and Dynamic Energy Budget parameters currently contains 92 species of turtles and 23 species of crocodiles. We discuss patterns of eco‐physiological traits of turtles and crocodiles, as functions of parameter values, and compare them with other taxa. Turtles and crocodiles accurately match the general rule that the life‐time cumulated neonate mass production equals ultimate weight. The weight at birth for reptiles scales with ultimate weight to the power 0.6. The scaling exponent is between that of amphibians and birds, while that for mammals is close to 1. We explain why this points to limitations imposed by embryonic respiration, the role of water stress and the accumulation of nitrogen waste during the embryo stage. Weight at puberty is proportional to ultimate weight, and is the largest for crocodiles, followed by that of turtles. These facts explain why the precociality coefficient, $s_{H}^{\mathrm{bp}}$—approximated by the ratio of weight at birth and weight at puberty at abundant food—decreases with ultimate weight. It is the smallest for crocodiles because of their large size and is smaller for turtles than for lizards and snakes. The sea turtles have a smaller $s_{H}^{\mathrm{bp}}$ than the rest of the turtles, linked to their large size and small offspring size. We link their small weight and age at birth to reducing risks on the beach. The maximum reserve capacity in both turtles and crocodiles clearly decreases with the precociality coefficient. This relationship has not been found that clearly in other taxa, not even in other reptiles, with the exception of the chondrichthyans. Among reptiles, crocodiles and sea turtles have a relatively large assimilation rate and a large reserve capacity.

## Graphical abstract

This paper discusses patterns in Dynamic Energy Budget parameters and implied traits that we found for turtles and crocodiles. We explain, for instance, why the proportionality of weight at birth with the square root of ultimate weight points to problems with water loss and nitrogen‐waste accumulation.

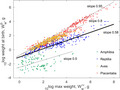

## 1 INTRODUCTION

Add‐my‐Pet (AmP) is an open access online collection of referenced data on animal energetics and Dynamic Energy Budget (DEB) parameters (AmP, 2021; Marques et al., 2018). The collection is run as a journal, meaning that everyone can contribute, and submissions are reviewed prior to acceptance. This study is part of a series of case studies on selected taxa from AmP whereby DEB parameters and associated traits are presented in eco‐evolutionary context. It focusses on traits of turtles (Testudines) and crocodiles (Crocodilia), using other reptiles as a reference; previous studies were on fish (Augustine et al., 2021; Kooijman & Lika, 2014; Lika et al., 2022), petrels and penguins (Kooijman, 2020), carnivores and pangolins (Kooijman & Augustine, 2022a), cephalopods (Kooijman & Augustine, 2022b).

Eco‐physiological traits are gaining more focus, as conservation physiology (*sensu* Cooke et al., 2013) is emerging as an ‘increasingly integrated and essential science’ (Cooke et al., 2013). Traits that are based on mechanistic models linking individuals to their environments can be used to predict how species respond to environmental change (Kearney et al., 2019), but also to study evolutionary drivers (Beekman et al., 2019; Jusup et al., 2017). Add‐my‐Pet (AmP) collection presents an array of such traits, and is therefore a most valuable resource.

Table 1 gives the number of reptile species currently included in the AmP collection, compared with the number of existing species. In our analysis and discussion, we use the Lepidosauria (= Rhynchocephalia + Squamata) and a dozen extinct reptile species (“dinosaurs”) as reference. Analysis is focused on turtles and crocodiles because we consider them `complete’ in the collection, that is, that it will be hard to find data on more species in open literature. The list of turtle and crocodile AmP species, the data types for each species and selected references can be found in the Appendix (Table A1 and Table A2).

**TABLE 1:** The number of reptile species in the AmP collection at time of the analysis (2022/04/04), the number of extant species (estimates from Wikipedia) and the coverage for reptile classes. Rhynchocephalia and Squamata form the class Lepidosauria, and are for simplicity presented as such in subsequent analysis

Taxon	AmP	Extant	Coverage
Testudines (turtles)	92	360	25.6%
Crocodilia (crocodiles)	22^a^	27	81.5%
Rhynchocephalia (tuatara)	1	1	100.0%
Squamata (snakes and lizards)	115	10,900	1.0%

^a^ Excluding the extinct *Deinosuchus rugosus* (terrible crocodile).

This paper first introduces turtles and crocodiles, briefly presents the Dynamic Energy Budget (DEB) framework used to formalize the traits, then discusses aspects of energetics and life history, and finalizes with a Discussion and conclusion section.

## 2 REPTILES, TURTLES AND CROCODILES

The extant “reptiles” are a polyphyletic group, with the 4 main lineages usually described as crocodilians, turtles, squamates (snakes and lizards), and tuatara. The name Reptilia is nowadays less frequently used, because it is not a clade (Shine, 2013). It should include birds, which, together with the crocodiles, form the clade Archosauria. Turtles and crocodiles are placed in the clade Archelosauria, while the “true” reptiles are a sister clade: the Lepidosauria (tuatara, lizards and snakes). Despite the exact grouping being still open to debate (Hedges & Poling, 1999), it is evident that reptiles have been independently evolving into very different animals since the Triassic (Hedges & Poling, 1999). We here focus on turtles (Testudines) and crocodiles (Crocodilia), but compare them with tuatara, squamates (Lepidosauria), and extinct reptiles present in the AmP collection (Pterosauria, Saurischia, Ornithischia, and Tyrannosauridae).

All turtles and crocodiles lay eggs, which, unlike many squamates which made the transition to ovovivipary, prevents them from living in cooler climates. Like most reptiles, they are ectothermic and master the art of regulating their body through behavior excellently. Interestingly, evidence exists for endothermy in the ancestors of the crocodiles, which converted back to ectothermy when adopting an aquatic life style (Seymour et al., 2004), and sea turtles are partially (Mrosovsky, 1980; Standora, 1982). Most turtles and all crocodiles have temperature dependant sex determination (Lee et al., 2019; Valenzuela & Adams, 2011), even though some turtles reverted to gene sex determination. The latter enables living in colder conditions, and is present also in all snakes. By contrast, the temperature‐dependant sex determination can also be found in some lizards, but not in habitats with extreme temperature fluctuations (Pen et al., 2010).

Some 60% of the turtle species are presently considered to be threatened (Rhodin et al., 2018), while of the 24 crocodile species, the IUCN crocodile specialist group lists 7 species as critically endangered and 12 species as vulnerable (IUCN‐Crocodile‐Specialist‐Group, 2021). The main threats, for turtles and crocodiles alike, are global climate change, habitat destruction, and illegal hunting, with (plastic) pollution as an emerging pressure for all wildlife, especially marine species such as sea turtles (Gall & Thompson, 2015; Marn et al., 2020; Nelms et al., 2016; Schuyler et al., 2014). Conservation in a changing world needs predictive mechanistic models (Wood et al., 2018), and functional traits derived from mechanistic models are invaluable in determining a species niche (Kearney & Porter, 2009). DEB theory has already been used to evaluate effects of climate change and plastic ingestion on sea turtles (Marn et al., 2020; Stubbs et al., 2017) and to optimize site selection for the western swamp turtle re‐introduction programs (Arnall et al., 2014, 2019), and to explain geographic shifts in reproductive patterns of a viviparous lizard (Schwarzkopf et al., 2016). We hope that this paper contributes to a better understanding of the eco‐physiology of turtles and crocodiles, and, in a much broader context, brings us closer to tackling major questions in ecology and evolutionary biology (Kearney et al., 2010).

## 3 DEB MODELS AND TRAITS

Dynamic Energy Budget models aim to quantify the various aspects of energy and mass budgets in dynamic environments in terms of temperature and food availability, throughout ontogeny, that is, embryo, juvenile, and adult. These aspects include food searching, feeding, defecation, digestion, storing, development, growth, reproduction, aging, and the fluxes of heat, CO_2_, H_2_O, O_2_ and N‐waste. Mass and energy conservation and stoichiometric constraints are respected explicitly. All parameters have a clear physical interpretation, and therefore simple dimensions. The standard (std) DEB model fits data for all turtle and crocodile species in the AmP collection very well; the median relative error for all data sets is 6%; this is also the median relative error for all 3000 species in the AmP collection.

The standard model is the simplest DEB model the other models that have been used in the AmP collection are one‐ or two‐parameter extensions to include, for example, larval development. The setup of the std model is as follows. A state of an individual is described by three state variables: maturity, $E_{H}$ (J)—that tracks the development of the individual but has no energy or mass, and two physical state variables—reserve, *E* (J), and structure, *V* (cm^3^ or g)—that determine the size of the individual. Food‐derived metabolites are first added to a reserve pool, and then reserve is mobilized for use in metabolism. Mobilization is such that weak homeostasis is respected: reserve density, that is, the ratio of the amounts of reserve and structure, does not change during growth in constant environments, possibly after an adaptation period. The rate of reserve mobilization depends on the amounts of reserve and structure and on the DEB parameter $\dot{v}$, energy conductance. A fixed fraction κ of the mobilized reserve is allocated to somatic maintenance and growth (soma), the rest to maturity maintenance and maturation (before puberty) or reproduction (after puberty). Feeding is taken to be proportional to squared length of structure, somatic maintenance to cubed length of structure, and maturity maintenance to the level of maturity. Reserve allocated to reproduction is collected in a reproduction buffer, with species‐specific buffer handling rules for the conversion to eggs. The growth‐trajectory of the std model simplifies to the von Bertalanffy (or better Pütter, see Kearney, 2020) growth model in constant environments. Pütter growth model, however, cannot handle dynamic environments (nor reproduction; Kearney, 2020), while the std model is designed for it. Ultimate length or weight and the von Bertalanffy growth rate are not parameters of the DEB model and depend on the environment, not only in reality, but also in DEB theory.

In the context of DEB theory, we define a trait as “a parameter or a function of parameters, which quantifies some eco‐physiological property of a species” (Kooijman et al., 2021). We followed the workflow that (measured) data from literature was used to estimate parameters, and these parameters are used to quantify the traits. So, traits here are not measured data, but instead model‐derived parameters and implied properties. Needless to say that the reliability of parameter values generally increases with data availability. The various AmP entries differ a lot in data availability, but in this way we could evaluate all traits for all species. Trait values for a species are interlinked; the strict application of mass and energy conservation rules in DEB theory contributes to this interlinking, and provides the consistency between traits.

Data and code used for parameter estimation are presented on the AmP website (AmP, 2021), together with references to the original literature, parameters, quantifiers for goodness of fit and data completeness. The site also presents a selection of eco‐physiological trait values for each species, as well as at the population level. All computations were performed using AmPtool and DEBtool (AmPtool, 2021; DEBtool, 2021)—two large computation packages supporting the AmP collection, which are freely available and can be used for further analysis.

### 3.1 Multidimensional scaling

Supplementary to analyzing distribution of traits and patterns in the co‐variation of parameter values, we have applied multidimensional scaling (MDS) on trait‐based distances between species (Kooijman et al., 2021). We chose 12 traits from those analyzed in this study (see Section 4.5 for a list of traits); a different set and/or number of traits could have been chosen (see Kooijman et al., 2021). The MDS needs a matrix of distances between species as input. The matrix is created based on the symmetric bounded loss function (Marques et al., 2019), which simultaneously takes into account all analyzed traits: the number of rows in the matrix corresponds to the number of species (here—243 species of reptiles), and the number of *relevant* columns depends on the eigenvalues: typically only the first few columns are relevant because the second eigenvalue is much smaller than the first one etc. By correlating each trait with (relevant) eigenvalues, it is possible to determine which traits contribute the most to the observed pattern among species. MDS was performed using the in‐built Matlab function cmdsc.m, and the correlation of trait distances with eigenvalues was performed using the DEBtool_M function corr.m. (Please see Kooijman et al., 2021 for presentation and examples of multidimensional scaling of animal traits in the context of DEB theory.)

## 4 ENERGETICS AND LIFE HISTORY

We first present the distribution of selected eco‐physiological traits for the turtles, crocodiles and Lepidosauria (squamates and tuatara), and then discuss some features in more detail. All temperature dependent traits are presented at a common reference temperature of 20°C.

### 4.1 Distributions of traits

**FIGURE 1:**
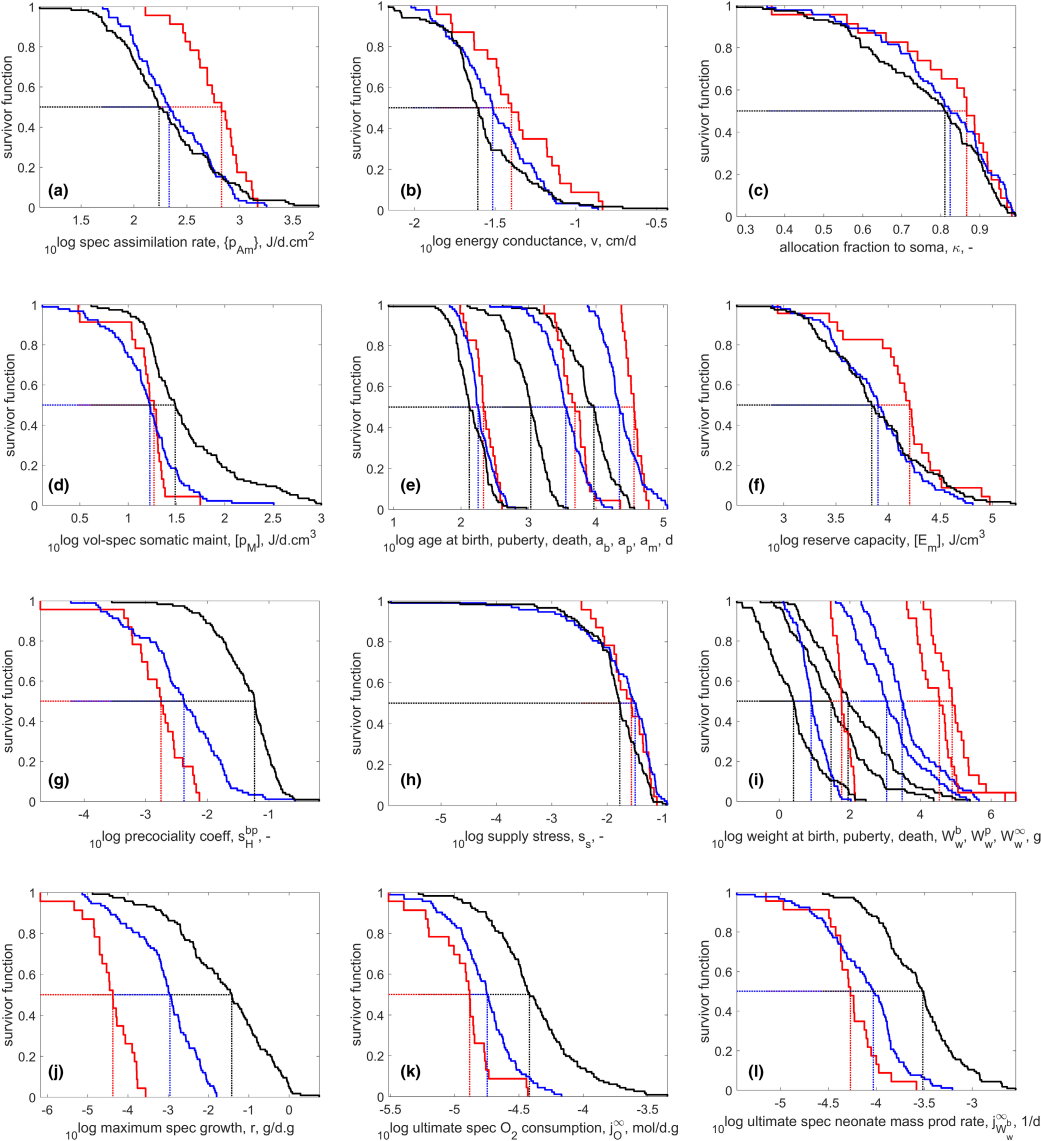
Survivor curves for selected DEB parameters and other traits for reptile taxa in the AmP collection: Testudines (blue), Crocodilia (red), Lepidosauria (black); for number of species see Table 1. Ages at birth, puberty and death are presented on the same plot; same for weights. All traits are presented for a body temperature of 20°C

Figure 1 shows survivor curves for selected traits, that is, for each trait the fraction of species for which the trait value exceeds the value on the abscissa. This is a very simple representation but can already point to general patterns and main differences or similarities between the groups. We here discuss the coherence.

The specific assimilation rate $\left\{{\dot{p}}_{\mathrm{Am}}\right\}$ of crocodiles is much larger than that of turtles and squamates (Figure 1a). This, combined with a smaller specific maintenance $\left[{\dot{p}}_{M}\right]$ (Figure 1d), explains in part why their ultimate weight is much larger (Figure 1i). See also Figure 4.

The energy conductance of turtles and crocodiles is quite a bit larger than that of squamates (Figure 1b). The effect of a large specific assimilation dominates that of a relatively large energy conductance in the maximum reserve capacity (Figure 1f), which equals the ratio of the two, and is the largest for crocodiles, implying they can sustain well the periods of starvation. An increase in energy conductance and in somatic maintenance both enhance growth. This is because the energy conductance determines the mobilization flux of reserve and the von Bertalanffy growth rate works out to be proportional to the specific somatic maintenance rate in the DEB context. (The specific growth rate at maximum growth turns out to equal 1.5 times the von Bertalanffy growth rate, see Kooijman et al., 2020.) Therefore, a large energy conductance combined with a small specific somatic maintenance can result in the same von Bertalanffy growth rate as vice versa. The effect of the energy conductance on growth is, however, more restricted, which explains why maximum specific growth is small in turtles and crocodiles (Figure 1j), despite their large energy conductance.

The allocation fraction to soma κ is similar in the three taxa, with the crocodiles having a slightly higher median value than the other two taxa (Figure 1c). This is in accordance with the highest ultimate weight of this class, as the ultimate size is proportional to κ (Lika et al., 2019).

A large energy conductance (Figure 1b) leads to a short incubation time, that is, smaller age at birth, but this is not what we observe (Figure 1e) because absolute egg size matters as well. Egg size is the largest for crocodiles, followed by that of turtles (Figure 1i).

The eggs and hatchlings of the crocodiles may be the largest among reptiles; however, they are relatively the smallest when the size of the parent is taken into account. This information is expressed as the precociality coefficient, which for crocodiles is lower than for turtles and much lower than for squamata (Figure 1g). The precociality coefficient, $s_{H}^{\mathrm{bp}}$, is a ratio of maturities at birth and puberty, but it roughly equals the ratio of the weights at birth and puberty at abundant food (Augustine et al., 2019). We will see that the weight at puberty is approximately proportional to ultimate weight, but that at birth scales with ultimate weight to the power 0.6. This implies that the differences in the precociality coefficient is mainly due to differences in adult weight.

**FIGURE A1:**
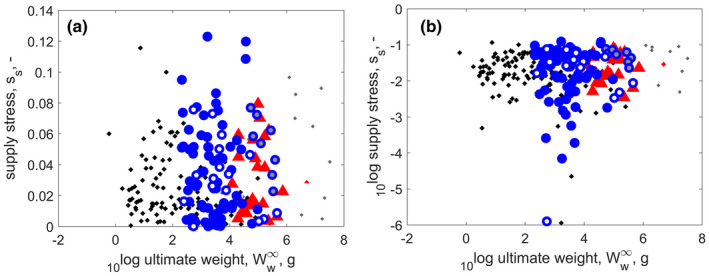
Supply stress for reptiles as function of ultimate weight (on a semi‐log scale on panel a, and log‐log scale on panel b) for: turtles (blue circles), crocodiles (red triangles), squamates and tuatara (black dots) and extinct reptiles (gray dots). Turtles show the largest range for this trait of the three reptile groups, implying a big diversity within this group: those living in the extreme conditions ‐ such as the desert serrated tortoise (*Psammobates oculiferus*) have a five times lower supply stress than those turtles living in freshwater ponds and rivers of temperate areas. The extremes are matched by a desert snake (*Psammophylax rhombeatus*) on the extreme supply‐end and mountain grasslizard (*Takydromus hsuehshanensis*) on the extreme demand‐end of the spectrum

The supply stress, $s_{s}$, is defined as maturity maintenance times squared somatic maintenance, divided by cubed assimilation and can take values between 0 and 4/27. It is similarly low for the three taxa (Figure 1h), meaning that they can rather easily deal with low food conditions and respond with low growth and reproduction (Lika et al., 2014). Birds and mammals have the highest supply stress, insects the lowest. Among reptiles, the median value is highest for turtles (0.0321), followed by that for crocodiles (0.0275), and then lepidosauria (0.0168). Sea turtles, perhaps due to their partial endothermy and generally relatively constant environments, have a higher median (0.0560) for this trait than other turtles. (See also Figure A1 in the Appendix).

Survivor curves for weight‐specific growth, respiration, and reproduction show that the crocodiles have the slowest metabolism among reptiles (Figure 1j–l), followed by turtles, then squamates. Low respiration (Figure 1k) comes with a long life span (Figure 1e), and a long live span compensates the low neonate mass production rate (Figure 1l), compared with the Lepidosauria. We come back to this in the discussion of Figure 3.

### 4.2 Respiration, life span, and reproduction

**FIGURE 2:**
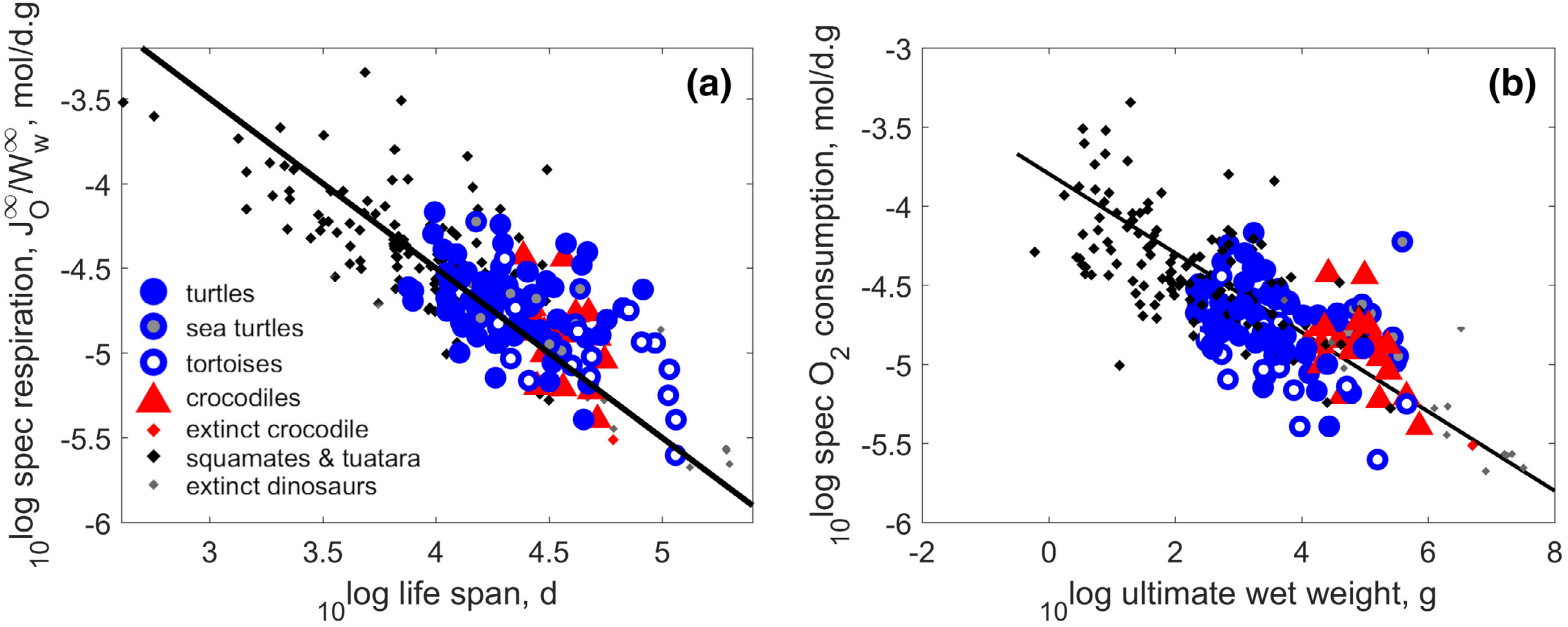
Panel a: The O_2_ consumption rate as function of life span. Panel b: The weight‐specific respiration as function of ultimate wet weight. The line in the panel a plot has a slope of −1, and the one in the panel b plot has a slope of −1/4. Lines were plotted without fitting. Markers: Blue dots represent 92 species of turtles (Testudines), with grey blue dots marking sea turtles (Chelonioidea) and empty blue dots tortoises (Testudinidae). Red triangles mark 22 species of living crocodiles (Crocodilia), and the extinct *Deinosuchus* is marked with a red dot. Black dots represent 115 species of squamates and tuatara (Lepidosauria), and grey dots a dozen extinct reptiles belonging to Pterosauria, Saurischia, Ornithischia, and Tyrannosauridae

**FIGURE 3:**
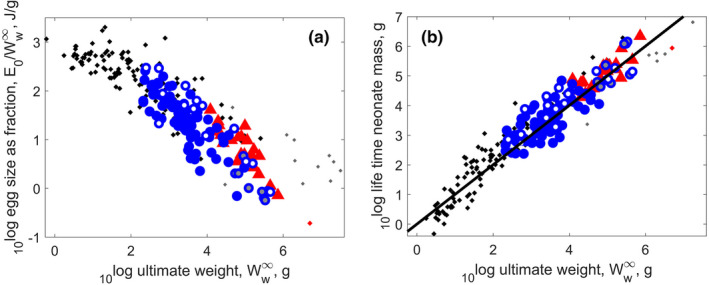
Panel (a): Egg size as fraction of ultimate weight decreases with ultimate weight. Panel (b): The life‐time cumulated neonate mass production increases with ultimate weight. Long life (Figure 2a), implying a long period of reproduction, offsets the relatively small egg size and offspring size of turtles and crocodiles. The line in panel b indicates equality, no parameters are involved. Markers as in Figure 2: turtles ‐ blue circles; crocodiles – red triangles; other reptiles – black dots

Respiration, life span, and reproduction are intimately connected for turtles and crocodiles (and other reptiles) (Figures 2 and 3), as found for chondrichthyans (Augustine et al., 2021) and for actinopterigyans (Lika et al., 2022). The relationships apply, with much more scatter, to all 3000 animal species in the AmP collection that covers all larger phyla (Augustine et al., 2021). The life span is inverse to the specific respiration (Figure 2a) and the life‐time cumulated neonate mass production equals the ultimate weight (Figure 3a). Long life, implying a long period of reproduction, offsets the relatively small egg size and offspring size of turtles and crocodiles (Figure 3b). We come back to the small egg size of turtles and crocodiles in the discussion. The lines shown in the figures have not been fitted to the data; no parameters involved.

Figure 2b shows that Kleiber's law also applies to reptiles, as explained by the physical co‐variation rules of DEB theory (Kooijman, 1986a, 2010). DEB theory does not work with allometric relationships. Specific respiration at abundant food works out as a cubic polynomial in ultimate length (Kooijman, 2010), but when curvature is ignored in a log‐log plot, the slope is close to −1/4, which is what we plotted in the plot (Figure 2b). The respiration of crocodiles, and the rather low one for turtles, fits the relationship well, meaning that their low respiration is mostly due to their large size. Body size is, in the context of DEB theory, an emergent property of metabolism, not an independent variable (Lika et al., 2019). So the figure shows how one function of DEB parameters relates to another function of these parameters.

### 4.3 Precociality coefficient and size at birth and puberty

**FIGURE A2:**
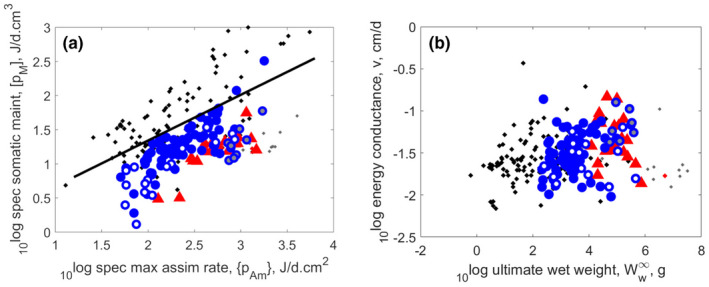
Panel (a): Volume specific maintenance rate, $\left[{\dot{p}}_{M}\right]$, as function of area‐specific maximum assimilation rate $\left\{{\dot{p}}_{\mathrm{Am}}\right\}$. Slope 2/3 is plotted in panel a, as the ration between surface area and volume of structure. Panel b: Conductance, $\dot{v}$, as function of ultimate wet weight

Size is, in large part, a result of the ratio between how much energy is assimilated and how much energy is left after maintenance needs have been met; turtles and crocodiles have relatively small maintenance costs relative to assimilation capacity, compared with other reptiles (Figure A2a in the Appendix). While some squamata are tiny, there are no very small turtles or crocodiles; the smallest living turtle is *Chersobius signatus* of 172 g; this is visible also in weight distribution Figure 1i.

**FIGURE 4:**
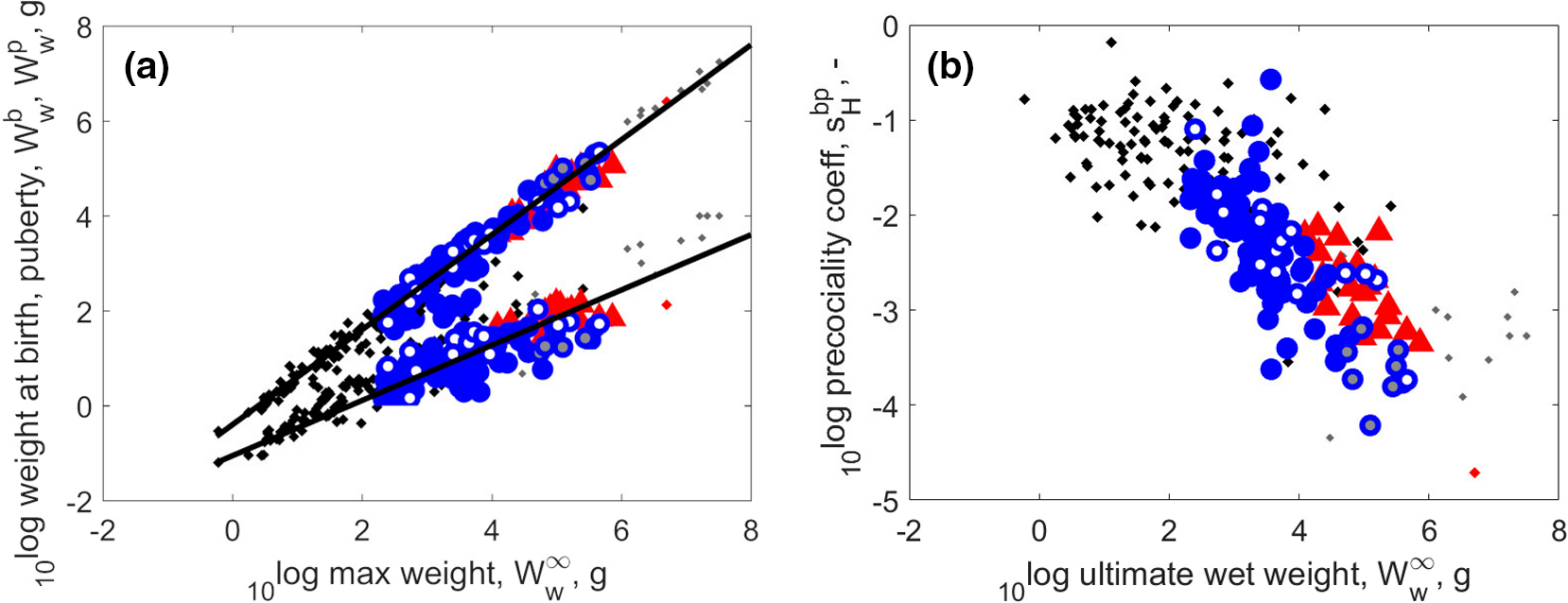
Panel (a): Weight at birth and at puberty as functions of ultimate weight. Panel (b): Precociality coefficient, $s_{H}^{\mathrm{bp}}$, as function of ultimate weight. Weight at puberty scales proportionally with ultimate weight (slope of 1), whereas weight at birth scales with a slope of 0.5818. The decrease of the precociality coefficient with ultimate weight follows from the previous scaling, since $s_{H}^{\mathrm{bp}}$ can be approximated by the ratio of weight at birth and weight at puberty. Markers as in Figure 2: turtles – blue circles; crocodiles – red triangles; other reptiles – black dots

Figure 4a shows that weight at puberty is directly proportional to ultimate weight (as expected by the physical co‐variation rules of DEB theory), with a fixed fraction 0.4. However, weight at birth scales to ultimate weight to the power 0.6, not only for turtles and crocodiles, but for all reptiles. Ratio of weight at birth and weight at puberty approximates to the precociality coefficient.

**FIGURE 5:**
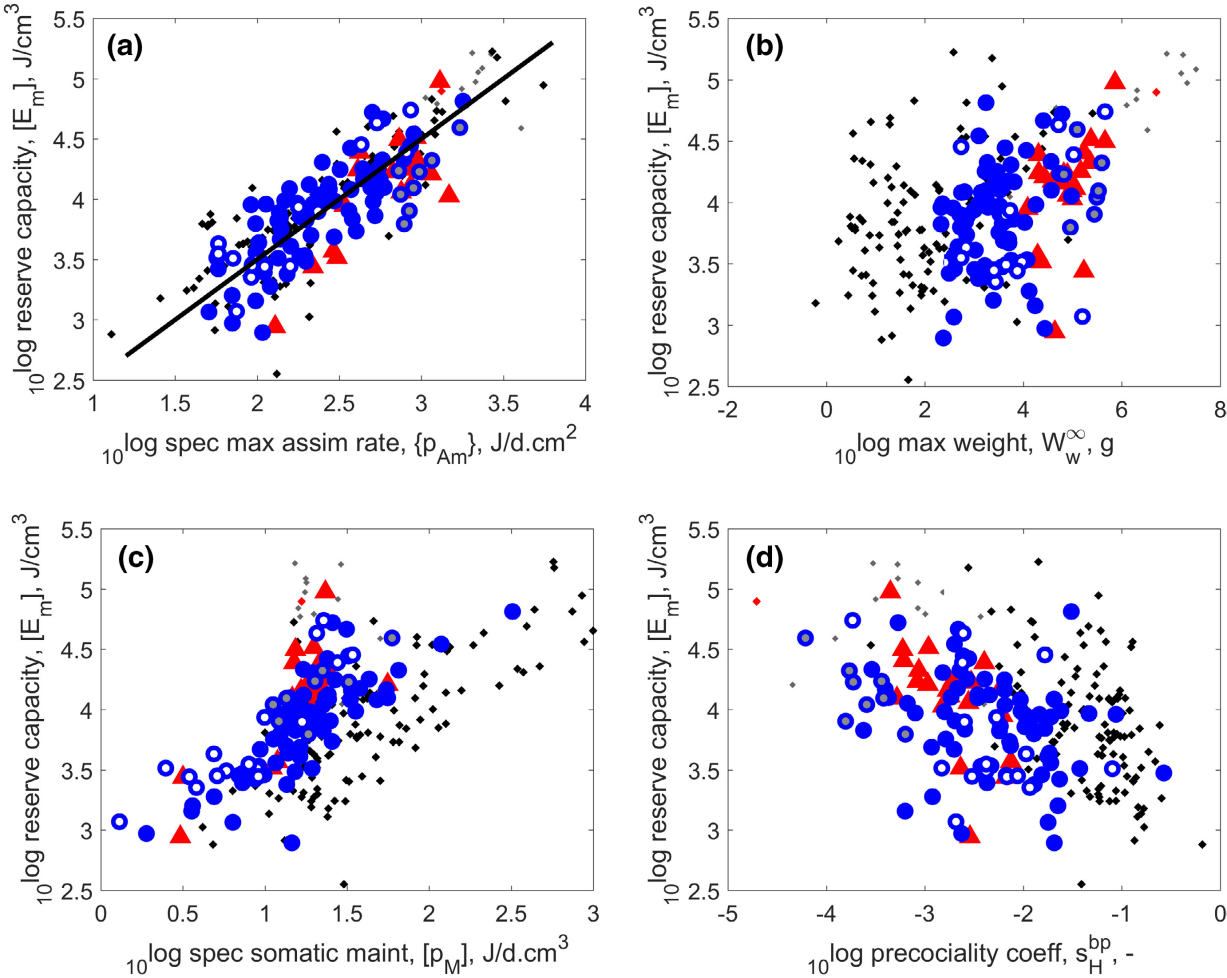
The maximum reserve capacity as functions of (Panel a) maximum specific assimilation rate; (Panel b) maximum weight; (Panel c) specific somatic maintenance rate, and (Panel d) precociality coefficient. The line in panel a indicates equality (slope of 1). Markers as in Figure 2: turtles – blue circles; crocodiles – red triangles; other reptiles – black dots. (The turtle outlier with the highest reserve capacity in all four plots is the Chinese pond turtle *Mauremys reevesii*)

**FIGURE A3:**
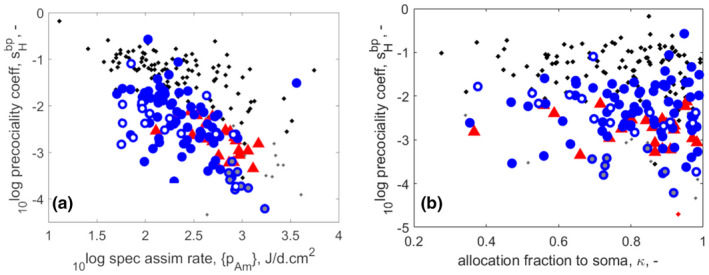
Panel (a): Precociality coefficient $s_{H}^{\mathrm{bp}}$ as a function of maximum specific assimilation rate $\left\{{\dot{p}}_{\mathrm{Am}}\right\}$. Panel (b): $s_{H}^{\mathrm{bp}}$ as function of allocation to soma κ (0<κ<1). There is substantial scatter in the traits, but lines could be drawn for illustration; slope between −0.5 and −0.6 fits well in panel a. There is no clear relationship between κ and $s_{H}^{\mathrm{bp}}$ for reptiles in general, except for tortoises (empty blue circles) where there seems to be a slight negative correlation. Even though crocodiles (red triangles) as a group have the lowest median precociality coefficient of all the reptiles (see also Figure 1), sea turtles (grey blue circles) have even lower values for $s_{H}^{\mathrm{bp}}$ than crocodiles

The physical co‐variation rules predict that the precociality coefficient roughly equals the weight at birth over that at puberty at abundant food, while the latter is more or less proportional to ultimate weight. We expect the precociality coefficient to scale with ultimate weight to the power −0.6, because birth weight was found to be proportional to ultimate weight to the power 0.6. This approximates what we did find (Figure 4b). The precociality coefficient is the smallest for crocodiles when classes are compared (Figure 1g), however, that of sea turtles is even smaller (see e.g., Figure 5d, and Figure A3 in the Appendix). The precociality coefficient quantifies how ‘immature’ an offspring is born, and is calculated as a ratio of maturity at birth and puberty. For reptiles, we can draw direct links to the egg size relative to adult size. We come back to this in the discussion.

### 4.4 Reserve capacity

Figure 5 shows (in sub‐figure a) that the maximum reserve capacity $\left[E_{m}\right]$ is proportional to the surface area‐specific assimilation rate $\left\{{\dot{p}}_{\mathrm{Am}}\right\}$; this is easy to understand since $\left[E_{m}\right]=\left\{{\dot{p}}_{\mathrm{Am}}\right\}/\dot{v}$. The physical co‐variation rules imply that $\left[E_{m}\right]$ is also proportional to maximum structural length, that is, to ultimate weight after some contribution of reserve is taken into account. This link, however, is not clearly visible for reptiles (Figure 5b). Maximum reserve capacity was found to increase with ultimate weight in chondrichthyans (Augustine et al., 2021), but not in actinopterigyans (Lika et al., 2022), which was explained by interference with the waste‐to‐hurry pattern (Kooijman, 2013). We do not think, however, that this pattern explains the lack of co‐variation between maximum reserve capacity and maximum weight here, since specific somatic maintenance $\left[{\dot{p}}_{M}\right]$ is too small to drive specific assimilation up, and the range for $\left[{\dot{p}}_{M}\right]$ is rather small for turtles and crocodiles. Energy conductance, $\dot{v}$—which is also affected in species with the waste‐to‐hurry pattern (Kooijman, 2013), and is the other parameter defining the $\left[E_{m}\right]$—has some scatter, but does not have a clear link to maximum weight (Figure A2b in the Appendix).

Maximum reserve capacity increases with specific somatic maintenance $\left[{\dot{p}}_{M}\right]$, Figure 5c, which is also part of the reason why the relationship between $\left[E_{m}\right]$ and ultimate weight is less clear: $\left[{\dot{p}}_{M}\right]$ reduces maximum structural length, so maximum weight. The ecological functionality of the co‐variation of maximum reserve capacity with specific somatic maintenance obviously helps to cope with temporary dips in food availability, although many turtle and crocodile species can enter torpor states.

Maximum reserve capacity tends to decrease with the precociality coefficient, $s_{H}^{\mathrm{bp}}$, although with considerable scatter (Figure 5d), which seems to be unique for turtles and crocodiles; we did not see this pattern before that clearly. The reason is probably that the scatter in the relative weights at birth and puberty is small (Figure 4a), so the signal is clear. We think that the existence of this pattern (Figure 5d) implies that $\left[E_{m}\right]$ in fact does increase with ultimate weight also for reptiles, but that the latter relationship comes out less clearly because more parameters contribute to ultimate weight, leading to a large scatter which obscures the signal.

### 4.5 Multidimensional scaling

We present results of multidimensional scaling (MDS) applied to reptiles for the following 12 eco‐physiological traits, most of them analyzed also in the previous sections: age at birth and puberty ($a_{b}$, $a_{p}$), life span ($a_{m}$), ultimate wet weight ($W_{w}^{\infty }$), reproduction rate at ultimate size ($R_{i}$), egg size ($E_{0}$), maximum reserve capacity ($\left[E_{m}\right]$), energy conductance ($\dot{v}$), volume‐specific maintenance rate ($\left[{\dot{p}}_{M}\right]$), area‐specific maximum assimilation rate ($\left\{{\dot{p}}_{\mathrm{Am}}\right\}$), supply stress ($s_{s}$), and precociality coefficient ($s_{H}^{\mathrm{bp}}$).

**FIGURE 6:**
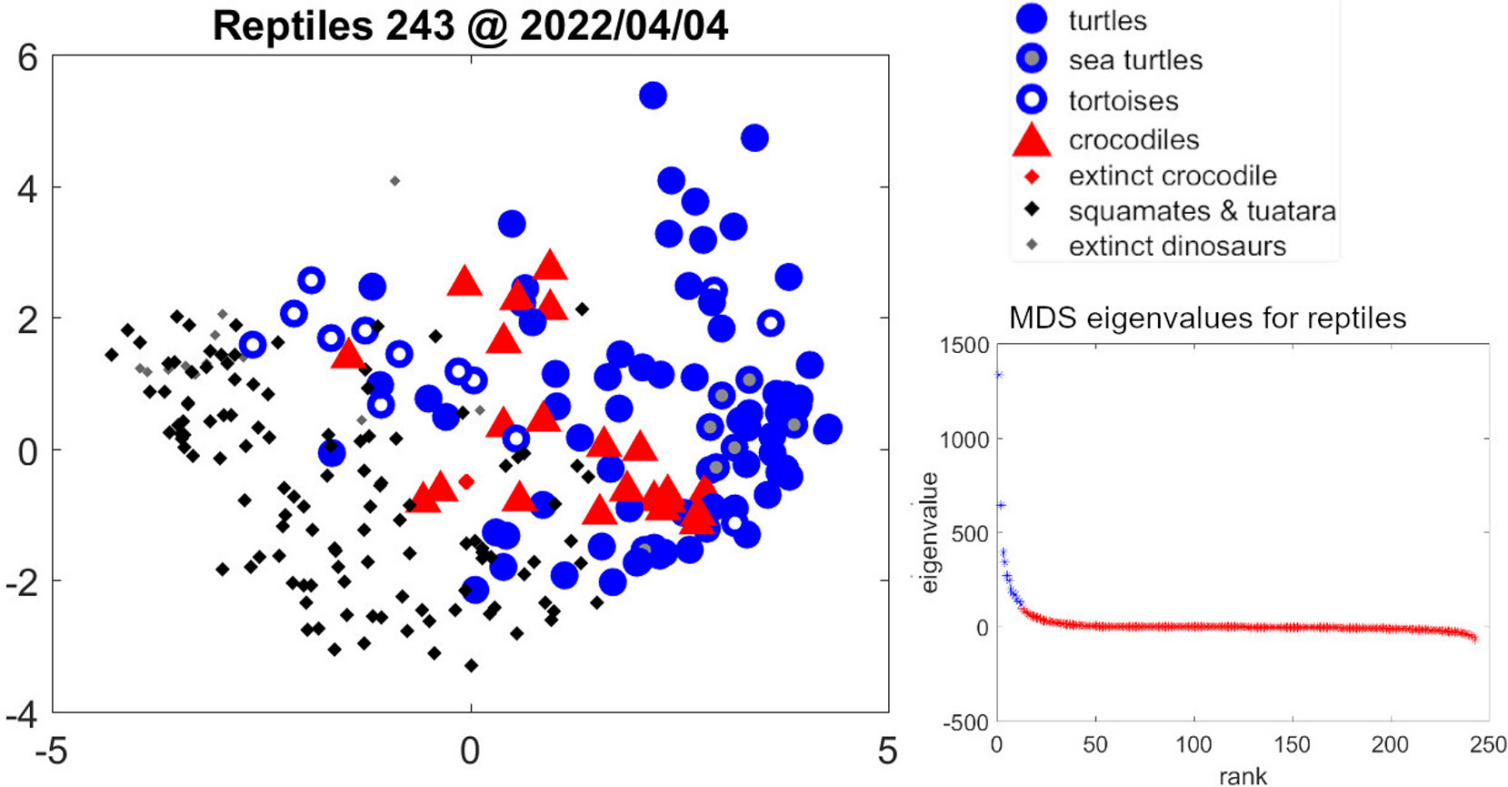
Multidimensional scaling applied to all 243 reptiles in the collection, using 12 arbitrarily chosen eco‐physiological traits (see text for list of traits). The bottom right figure presents all eigenvalues. The first 12 eigenvalues are presented in blue. Markers: Blue dots represent turtles (Testudines), with grey blue dots marking sea turtles (Chelonioidea) and empty blue dots tortoises (Testudinidae). Red triangles mark living crocodiles (Crocodilia), and the extinct *Deinosuchus* is marked with a red dot. Black dots represent squamates and tuatara (Lepidosauria), and grey dots a dozen extinct reptiles belonging to Pterosauria, Saurischia, Ornithischia, and Tyrannosauridae

Multidimensional scaling clusters species in multidimensional space. We present here “only” a two‐dimensional plot (Figure 6), but the eigenvalues in the bottom right corner of the figure indicate that the first two dimensions are the most relevant ones (third eigenvalue is already much smaller than the first and the second one; Figure 6), and so the 2D‐graph is a good indication of the species’ position relative to each other. As a general pattern, we can observe that crocodiles cluster together, as do most of the turtles. Within the turtle group, sea turtles form a clear subgroup, as do most of the tortoises (Figure 6). Relative to the x‐axis (representing the first eigenvector), we can observe a transition between the Lepidosauria (squamates + tuatara) on the left, then Testudinidae (tortoises) and crodociles (Crocodilia) in the middle, and then remaining turtles (Testudines), with sea turtles (Chelonioidea) close to the far right (Figure 6).

When correlating the traits with the first and second eigenvector, we see that the life span and age at puberty have the highest (−ve) correlation with the first eigenvector, followed by the (+ve) precociality coefficient (correlation coefficients larger than 0.7, 0.6, and 0.5, respectively). Maximum reserve capacity, somatic maintenance, and maximum assimilation have the highest (+ve) correlation with the second eigenvector (correlation coefficients larger than 0.5). This points to the main traits characterizing the analyzed groups, as we discuss in the following section.

## 5 DISCUSSION AND CONCLUSIONS

Reptiles are a diverse polyphyletic group, but, as we have just shown, their eco‐physiological traits also point to similarities in trait patterns, and coherence within and between groups. Multidimensional scaling (MDS) on trait‐based distances between species supplements our efforts to find patterns in the co‐variation of parameter values. We used most of the traits analyzed in this study (see Section 4.5 for a list of traits) to expand on the turtle‐focused MDS presented in Kooijman et al. (2021). Results of the MDS analysis corroborate the grouping evident already in the simple co‐variation analysis: in the multidimensional space crocodiles again cluster together, as do the turtles, both of them separate from the rest of the reptiles. Within turtles, sea turtles and tortoises form separate clusters (Figure 6).

When using this specific selection of traits and correlating them to the first two eigenvectors, we can identify main characteristics (i.e., eco‐physiological traits) which place species at either of the two extremes: at one of the extremes we have slow‐maturing, long‐living, relatively large individuals with relatively small offspring (i.e., a small precociality coefficient) and relatively high metabolism, but also good ability to withstand food shortages (high reserve capacity)—such as sea turtles. At the other extreme, we have individuals with a relatively fast life cycle, and with offspring size more similar to parent size (i.e., a higher precociality coefficient), which are less tolerant to periods of starvation (i.e., they have a lower maximum reserve capacity)—such as lizards and snakes. This points to quite specific environmental pressures, and is therefore encouraging that related species experiencing similar environments cluster together.

Even though (ultimate) weight is *not* one of the traits with a strong correlation to one of the two axes in the MDS plot, the results section shows that it does have a strong relationship to many eco‐physiological traits. Coupling of many eco‐physiological traits to size (Calder, 1984; Peters, 1983) has well understood reasons (Kooijman, 2010); the fact that large weight allows for long starvation intervals and dives (for aquatic species) is very relevant in this context. Moreover, both turtles and crocodiles—frequently among the largest reptiles—easily switch to a estivation/torpor/hibernation state where they further reduce their maintenance costs (Hochscheid et al., 2007; Nussear et al., 2007; Staples, 2016).

Generally, crocodiles as a group have the slowest metabolism among reptiles (Figures 1 and 2), but their low respiration is matched—or even exceeded—by low respiration of large and long lived tortoises and sea turtles (Figure 2). Maximum specific growth rates of turtles are larger than that of crocodiles and smaller than that of other reptiles (Figure 1j), but there is much variation within the group (not shown): sea turtles (Chelonioidea) have a relatively large maximum specific growth rate, but their close relatives, the mud and musk turtles (Kinosternidae) have a relatively small maximum specific growth rate, a small ultimate weight and typical relative weight at birth. This seems to reflect opposing selection pressures within the Chelydroidea (Chelonioidea + Kinosternidae).

Specific respiration of turtles and crocodiles (as well as other reptiles) is inverse to their life span (Figure 2a), and life‐time cumulative neonate mass production equals ultimate weight (Figure 3b); a pattern also observed in fish (Augustine et al., 2021; Lika et al., 2022). In some reptile groups—such as sea turtles, and larger crocodiles and tortoises—the eggs and offspring are small relative to ultimate weight (Figure 3a). The fact that the equality between life‐time cumulative neonate mass and ultimate weight holds also for these groups, suggests that the small offspring size is offset by a large number of offspring throughout the reproductive period. We discuss later the possible explanation for having such small offspring.

For both turtles and crocodiles (and reptiles in general), weight at puberty is directly proportional to ultimate weight, but the weight at birth as a fraction of ultimate weight decreases with ultimate weight substantially (Figure 4a). This calls for an explanation, and we do it in the context of other vertebrates: amphibia, birds, and mammals, but also fish.

**FIGURE 7:**
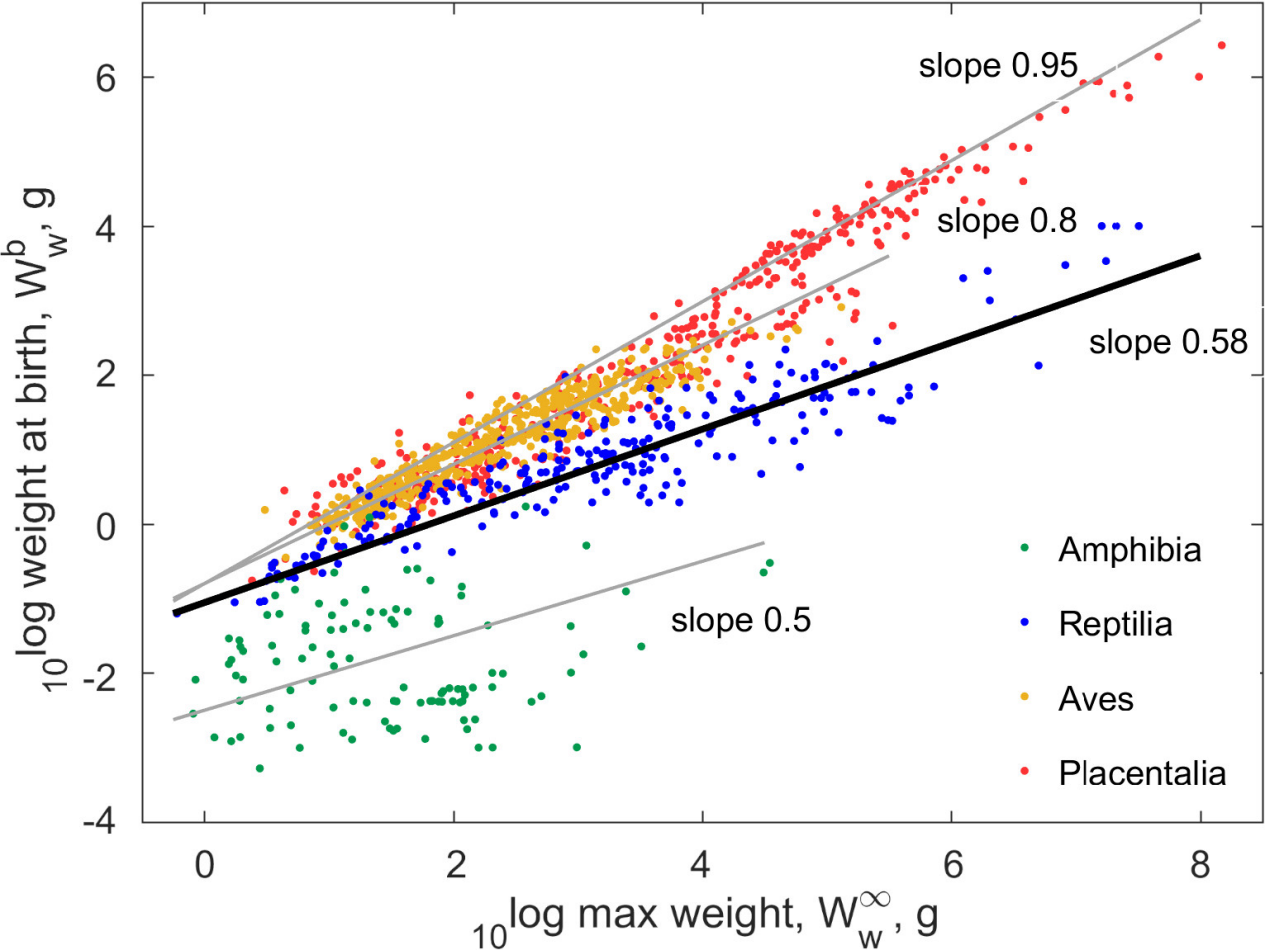
Scaling exponent for weight at birth as a function of ultimate weight for amphibia, reptiles, birds, and mammals (Modified from Augustine et al., 2021). Size at birth (and therefore egg size) increases with ultimate weight, but less so for reptiles than for birds and mammals. We discuss this in the text

Figure 7 presents the behavior of the scaling exponent for weight at birth as a function of ultimate weight, for vertebrates that live on land. We focus on this scaling exponent because constraints of the type that we will consider become more apparent for increasing size. Birds have a scaling exponent of 0.8 (Augustine et al., 2021), while their eggs—directly proportional to size at birth—are relatively larger than that of reptiles. Although the body size‐range for birds is smaller than that of reptiles, the smaller scaling exponent for reptiles is probably not due to mechanical constraints of producing large eggs; the 3.9 kg kiwi has an egg size of even 20% of its body weight, implying that larger birds could lay larger eggs too. This view is confirmed by the exponent of placentalia of 0.946 (Augustine et al., 2021), which produce neonates of similar relative size compared to birds, so larger than that of reptiles, while their range of body sizes exceeds that of reptiles.

This points to explanations other than mechanical constraints: (i) limitation of respiration during the embryo stage, (ii) the accumulation of nitrogen waste in the egg, and (iii) water loss from the egg. The placentalia escaped these problems by placental vivivary.

Dioxygen limitation was already suggested for amphibia, which produce aquatic eggs with jelly envelopes that might reduce transport of O_2_ (Seymour & Bradford, 1995); they have a scaling exponent of 0.5 (Augustine et al., 2021), so somewhat smaller than the reptiles. The biggest amphibians, i.e. the giant salamanders *Andrias* with the largest eggs, live in cold water, where respiration limitation is weaker due to low metabolic needs and high solubility of O_2_ in cold water, and the produced nitrogen waste can easily dissipate. The nitrogen waste of amphibians is mainly ammonia in tadpoles, which is toxic, but they hardly suffer from this in an aquatic environment where ammonia can easily dissipate. Many chondrichthyans sport vivipary and their metabolic rate is less then that of birds, have relatively large neonates and a scaling exponent of 0.88 (Augustine et al., 2021), between that of birds and placentalia. This suggests that they too escaped the selection pressure from oxygen limitation.

Terrestrial environments exert a strong selective pressure on water loss and nitrogen waste accumulation in eggs. Birds and reptiles are uricoletic (Withers, 1992), so they solved the nitrogen waste problem by making use of non‐solvable (so non‐toxic), but energetically expensive types of nitrogen waste. Birds have much higher metabolic rates than reptiles and use lipids as energy source, which give much more water than proteins when oxidized during metabolism. This allowed birds to insert larger pores in their egg shells, compared to reptiles, increasing the O_2_ availability without loosing too much water. By contrast, reptiles primarily use proteins as energy source. They, therefore, need to preserve water in eggs better than birds, which they do by having smaller pores in egg shells, limiting O_2_ availability and thus maximum egg size. Altricial birds that nest in trees show that water loss is an important issue; they hatch with extra water content in their tissues which reduces till fledging (Augustine et al., 2019; Konarzewski, 1988). This illustrates the conflicting needs of water and dioxygen transport for terrestrial eggs, and points to the conclusion that birds managed to escape these problems almost completely, in view of their scaling exponent being close the one, like was found for weights at puberty for all vertebrate taxa.

Relatively small eggs (and offspring) of some turtles and crocodiles (Figure 3a) could be linked to specific ecological pressures. Turtles and crocodiles make nests and bury their eggs in sand, where temperature depends on sunshine, or in a heap of dead leaves, where temperature depends on fungal activity. Incubation is timed when environmental conditions are favorable, and so the longer the incubation lasts—incubation duration increasing with egg size—the more difficult it becomes to select the proper time window, and the higher the risk of nest destruction. Shorter incubation times are also incentivized by the fact that nests are extremely vulnerable to predation, sea turtles being the prime example (Bolten et al., 2011; Whiting & Whiting, 2011). Although sea turtles have parameters in the range of other turtles, within this range they have one of the smallest relative weight and age at birth, typical weight at puberty, and their ultimate weight is at upper end of the turtle range (Figure 4). Large adult size corresponds to a large reproductive output. As a consequence of eggs being small, the number of eggs is relatively large (Figure 3); see also Beekman et al. (2019). We suggest that their small eggs and short incubation times are adaptations to minimize their stay on land to reduce the risks of flooding (Ewert, 1979), and predation. The latter interpretation is further supported by synchronized hatching, not only within a nest, but also between nests on the same beach. Details of beach conditions seem very important to the turtles, since the selection of nesting sites has a strong historic component which explains most of their long‐distance migration behavior. Crocodiles have the same problem of very vulnerable early life stages, but solved it in a different way: by guarding their nest with a respectable set of teeth and substantial body mass. Their relative weights at birth and puberty are typical, but their ultimate mass is at the upper end of the range for the Archelosauria. For comparison, the exponent for oviparous and viviparous chondrichthyans is the same, which suggests that reduction of predatory risks by reducing eggs size, thus shortening incubation time, might be less important for chondrichthyans (Augustine et al., 2021).

The comparison of life history traits between taxa is not without problems; it matters a lot how we compare exactly and what is taken as reference. For instance, when we suggest that dioxygen availability or toxicity of accumulated nitrogen waste limit embryo size, we do not imply that the embryo actually experiences such limitation or toxic effects, only that egg size is such that these problems are avoided. The large literature on bird egg development stresses the role of O_2_ limitation (Hoyt & Rahn, 1980; Tazawa et al., 1983; Visschedijk, 1968; Visschedijk & Rahn, 1983). The authors point that the maximum flux through the pores is egg‐size independent, from hummingbird to ostrich, and point to the levelling of dioxygen consumption prior to pipping. This implies that O_2_ is actually limited. If true, we disagree with this view. The constancy of maximum dioxygen flux through the pores is taken as a consequence of the need to minimize water loss: pores should not be larger than strictly necessary. The levelling of dioxygen consumption prior to hatching also occurs in very different species that do not have an egg shell (Kooijman, 1986b), and therefore cannot be caused by the limiting O_2_ flux. DEB theory takes this as a result of depleting reserve, which not only causes a levelling of, but even a decline of dioxygen use prior to hatching, as is really clear in eggs of the pig‐nosed turtle, *Carettochelys insculpta*, and the Australian freshwater crocodile, *Crocodylus johnsoni* (Zonneveld & Kooijman, 1993), where embryos delay hatching by waiting for their nest mates to be ready for synchronous hatching.

Coherence and consistency are crucial conditions for comparing eco‐physiological traits within and between taxa, and we believe that using DEB model‐derived traits greatly adds to both of these prerequisites (Kooijman et al., 2021). Furthermore, it bypasses the data limitations which are often imposed when a broader (or more in‐depth) analysis is required (Wood et al., 2018), because (i) DEB models need relatively few data to parameterize (Marques et al., 2018), and (ii) all traits can be computed for all species for which DEB parameters have been estimated, which is currently over 3000 animal species (AmP, 2021). Analyzing trait patterns then further improves the process of parameter estimation for a species of interest, resulting in a better predictions and more in‐depth knowledge about the species. Knowledge about metabolic performance under various external and internal pressures is key to conservation biology, sustainable management and environmental risk assessment, which are seen as interlinked fields with much to gain from coherent and applicable predictive models (Wood et al., 2018).

## AUTHOR CONTRIBUTIONS

**Nina Marn:** Data curation (equal); Investigation (equal); Validation (equal); Writing—review and editing (equal). **Sebastiaan A. L. M. Kooijman:** Conceptualization (equal); Data curation (equal); Formal analysis (equal); Investigation (equal); Software (equal); Writing—original draft (equal).

## CONFLICT OF INTEREST

The authors declare no conflict of interest.

## Data Availability

The underlying data come from published literature. The data and the references to where it comes from can be found on the Add‐my‐Pet website https://www.bio.vu.nl/thb/deb/deblab/add_my_pet as well as on its mirror at https://debtheory.fr/add_my_pet/. There you can also find the code that has been used to estimate parameter values for each species. This code uses the software packages AmPtool AmP, 2021 and DEBtool DEBtool, 2021, which are freely available via Github: https://github.com/orgs/add‐my‐pet/repositories. A selection of references to data for each species is also given in the Appendix.

## 1

**TABLE A1:** Testudines and Crocodilia species that are included in the AmP collection at 2021/10/02, the data types as extracted from the literature and selected references. Data were also obtained from websites, which are presented in the AmP website for each entry. The codes of the data types are presented in Table A2

Species	Data	References
Actinemys marmorata	am, Lp, Li, Wwb, Wwi, Ri, t‐L	Germano and Riedle (2015)
Aldabrachelys gigantea	ab, ap, am, Lb, Lp, Li, Wwb, Wwp, Wwi, Ri	Ernst and Barbour (1989)
Alligator mississippiensis	ab, ap, am, Lp, Li, Ww0, Wwi, Ri, t‐L	Deeming and Ferguson (1991), Jacobson and Kushlan (1989)
Alligator sinensis	ab, ap, am, Lp, Li, Ww0, Wwb, Wwi, Ri, t‐L, t‐Ww	Herbert et al. (2002)
Apalone mutica	am, Lp, Li, Wwb, Wwi, t‐L, L‐N	Plummer (1977)
Apalone spinifera	ab, ap, am, Lb, Lp, Li, Wwb, Wwi, Ri, t‐L, L‐dL	Plummer and Mills (2015)
Astrochelys yniphora	ab, am, Lb, Lp, Li, Wwp, Wwi, Ri, L‐dL	Smith et al. (2001)
Batagur affinis	ab, ap, am, Lb, Lp, Li, Ww0, Wwb, Wwi, Ri, t‐Ww, t‐L	Hairul and Shahrul Anuar (2014), Moll et al. (2015)
Batagur baska	ab, ap, am, Wwb, Wwi, Ri, t‐Ww	Weissenbacher et al. (2015)
Caiman crocodilus	ab, ap, am, Lb, Lp, Li, Ww0, Wwi, Ri, t‐L	Campos et al. (2008), Miranda et al. (2002), Mourao et al. (2014)
Caiman latirostris	ab, ap, am, Lp, Li, Wwb, Wwi, Ri, t‐L	Viotto et al. (2020)
Caiman yacare	ap, am, Lb, Lp, Li, Wwb, Wwi, Ri, t‐L	Mourao et al. (2014)
Caretta caretta	ah, ab, ap, am, Lh, Lb, Lp, Li, Wwh, Wwb, Wwp, Wwi, Ri, E0, T‐ah, t‐L_T, t‐Ww_T, L‐Ww, L‐N, L‐dL, L0‐Lt	Bjorndal et al. (2000), Bjorndal et al. (2013), Braun‐McNeill et al. (2008), Byrd et al. (2005), Ehrhart and Yoder (1978), Godfrey and Mrosovsky (1997), Hawkes et al. (2005), Hays and Speakman (1991), Hildebrand and Hatsel (1927), Miller et al. (2003), Norton (2005), Parker (1926, 1929), Reich et al. (2008), Scott et al. (2012), Snover et al. (2007), Spotila (2004), Stokes (2014), Stokes et al. (2006), Stoneburner (1980), Tiwari and Bjorndal (2000), Tucker (2010), Wabnitz and Pauly (2008), Zug et al. (1986)
Caretta caretta MED	ah, ab, ap, am, Lb, Lp, Li, Wwb, Wwp, Wwi, Ri, E0, T‐ah, t‐L_fT, t‐Ww_T, L‐Ww, L‐N	Broderick et al. (2003), Casale et al. (2011, 2009), Cateau (2014), Godfrey and Mrosovsky (1997), Groombridge (1990), Hays and Speakman (1991), Margaritoulis et al. (2003), Marn et al. (2019), Piovano et al. (2011), Reid et al. (2009), Stokes (2014), Tiwari and Bjorndal (2000), Zbinden et al. (2006)
Carettochelys insculpta	ap, am, Lb, Lp, Li, Wwb, Wwp, Wwi, Ri, t‐WwVe, t‐JOe, t‐WwYe	Doody et al. (2003), Webb et al. (1986)
Centrochelys sulcata	ap, am, Wwb, Wwi, Ri, t‐Ww, L‐Ww	Ritz et al. (2010)
Chelodina oblonga	ab, ap, am, Lb, Lp, Li, Wwb, Ri, L‐dL, t‐L, L‐Ww	Ernst and Barbour (1989), Kennett (1996)
Chelonia mydas	ah, ab, ap, am, Lh, Lp, Li, Wwh, Wwp, Wwi, Ri, E0, T‐ah, t‐WwYe_T, t‐WwVe_T, t‐JOe_T, t‐JCe_T, L0‐Lt, L‐Ww	Balazs and Chaloupka (2004), Balazs and Ross (1974), Bell et al. (2005), Bjorndal and Carr (1989), Broderick et al. (2003), Chaloupka et al. (2004), Christens (1990), Ekanayake et al. (2016), Frazer and Ehrhart (1985), Frazer and Ladner (1986), Goshe et al. (2010), Guinea (2009), Hendrickson (1958), K.S. et al. (2014), Limpus (1993), Limpus and Fien (2009), Limpus and Nicholls (1988), Limpus et al. (2005), Moreira et al. (1995), Pereia et al. (2011), Prince (2017), Rusli et al. (2016), Salmon et al. (2009), Troeng and Chaloupka (2007), Venkatesan et al. (2005), Wine (2016), Zurita et al. (2012)
Chelonoidis niger	ab, ap, am, Lb, Li, Wwb, Wwi, Ri, t‐Ww	Ritz et al. (2010)
Chelus fimbriata	ab, am, Lb, Lp, Li, L_t, Wwb, Wwi, Ww_t, Ri, t‐L	Prithard (2008)
Chelydra serpentina	ap, am, Lp, Li, Wwb, Wwi, Ww_L, Ri, t‐Ww, T‐a_b	Williamson et al. (1989), Yntema (1978)
Chrysemys picta	ab, ap, am, Li, Wwb, Ri, t‐L, t‐Ww	Rowe (1994), Wilbur (1975)
Claudius angustatus	ab, ap, am, Lb, Lp, Li, Wwb, Wwp, Wwi, Ri	Legler and Vogt (2013)
Clemmys guttata	ab, ap, am, Lb, Lp, Li, Wwi, Ri, t‐L	Ernst (1975)
Crocodylus acutus	ab, ap, am, Lb, Lp, Li, Wwb, Wwi, Ri, L0‐Lt, L‐Ww	García‐Grajales et al. (2012)
Crocodylus intermedius	ap, am, Lb, Lp, Li, Wwi, Ri, t‐L	Seijas (2016)
Crocodylus johnsoni	ab, ap, am, Lb, Lp, Li, Wwb, Wwp, Wwi, Ri, t‐WwYe, t‐WwVe, t‐JOe	Whitehead (1987), Whitehead et al. (1990)
Crocodylus mindorensis	ab, ap, am, Lp, Li, Wwb, Wwp, Wwi, Ri	Marzola et al. (2014)
Crocodylus moreletii	ab, ap, am, Lb, Lp, Li, Wwb, Wwi, Ri, L0‐Lt, L‐Ww	Pérez‐Higareda et al. (1995)
Crocodylus niloticus	ab, ap, am, Lb, Lp, Li, Wwb, Wwp, Wwi, Ri, L‐Ww	Ngwanya et al. (2013)
Crocodylus palustris	ab, ap, am, Lb, Lp, Li, Wwb, Wwi, Ri	Brien (2015)
Crocodylus porosus	ab, ap, am, Lb, Lp, Li, Wwb, Wwp, Wwi, Ri, L‐Ww	
Crocodylus rhombifer	ab, ap, am, Lp, Li, Wwb, Wwi, Ri	Targarona et al. (2010)
Crocodylus siamensis	ab, ap, am, Lb, Lp, Li, Wwi, Ri, L‐Ww	Chentanez et al. (1983), Kanwatakid‐Savini et al. (2012)
Cuora flavomarginata	ab, ap, am, Lb, Lp, Li, Wwb, Wwp, Wwi, Ri, t‐L	Chen and Lue (2002)
Deinosuchus rugosus	ap, am, Li, Wwi, Ri, t‐L	Erickson and Brochu (1999)
Deirochelys reticularia	ab, ap, am, Lb, Lp, Li, Wwb, Wwi, Ri, t‐L	Buhlmann et al. (2009)
Dermatemys mawii	ab, ap, am, Lb, Lp, Li, L_t, Wwb, Wwp, Wwi, Ww_t, Ri	Legler and Vogt (2013)
Dermochelys coriacea	ab, ap, am, Lb, Lp, Li, Wwb, Wwi, Ri, JXi, pAi, t‐L_f, t‐Ww	Jones (2009)
Elseya albagula	ab_T, ap, am, Lb, Lp, Li, Ww0, Wwi, Ri, t‐L	Limpus (2008)
Elseya dentata	ab, ap, am, Lb, Lp, Li, Wwb, Wwp, Wwi, Ri, L‐dL, t‐L	Ernst and Barbour (1989), Kennett (1996)
Elusor macrurus	ab, ap, am, Lb, Lp, Li, Wwi, Ri, t‐L	Limpus (2008)
Emydoidea blandingii	ab, ap, am, Lb, Li, Wwb, Wwi, Ri, t‐L, t‐Ww	Congdon and van Loben Sels (1991)
Emydura macquarii	ab_T, ap, am, Lb, Lp, Li, Wwb, Wwp, Wwi, Ri, t‐L	Spencer (2002)
Emydura victoriae	ab, ap, am, Wwb, Wwi, Ri, t‐Ww	Gaikhorst et al. (2011)
Emys orbicularis	ab, ap, am, Lb, Lp, Li, Wwb, Ri, t‐L, t‐Ww	Masin et al. (2015)
Eretmochelys imbricata	ab, ap, am, Lb, Li, Wwb, Wwi, Ri, t‐L	Bell and Pike (1980), Witzell (1980)
Gavialis gangeticus	ab, ap, am, Lb, Lp, Li, L_t, Ww0, Wwb, Wwi, R_L	
Geochelone elegans	ab, ap, am, Lb, Li, Ww0, Wwb, Wwi, Ri, t‐Ww, t‐L	Vyas (1997)
Glyptemys insculpta	ab, ap, am, Lb, Lp, Li, Wwb, Wwi, Ri, t‐L	Marchand et al. (2018)
Glyptemys muhlenbergii	ab, ap, am, Lb, Lp, Li, Wwi, Ri, t‐L	Lovich et al. (1998)
Gopherus agassizii	ab, ap, am, Lb, Lp, Li, Wwb, Wwp, Wwi, Ri, t‐L	Ernst and Barbour (1989), Medica et al. (2012)
Gopherus berlandieri	ab, ap, am, Lb, Li, Wwb, Ri, t‐Ww, t‐L	Judd and McQueen (1980)
Gopherus morafkai	ab, ap, am, Lb, Lp, Li, Wwb, Wwi, Ri, t‐L	Averill‐Murray et al. (2018), Bridges (2012)
Gopherus polyphemus	ab, ap, am, Lb, Lp, Li, Wwb, Wwp, Wwi, Ri, t‐L	Ernst and Barbour (1989), Mushinsky et al. (1994)
Graptemys caglei	ab, ap, am, Lb, Li, Wwi, Ri, t‐L	Lindeman (1999)
Graptemys ernsti	ab, ap, am, Lb, Li, Wwi, Ri, t‐L	Lindeman (1999)
Graptemys oculifera	ab, ap, am, Lb, Li, Wwb, Wwi, Ri, t‐L	Jones and Hartfield (1995)
Graptemys ouachitensis	ab, am, Lb, Lp, Li, Wwi, Ri, t‐L	Lindeman (1999)
Graptemys pseudogeographica	ab, am, Lb, Lp, Li, Wwi, Ri, L‐r	Webb (1961)
Graptemys versa	ab, ap, am, Lb, Lp, Li, Wwi, t‐L, L‐N	Lindeman (2005)
Heosemys spinosa	ab, am, Lb, Lp, Li, Ww0, Wwb, Wwi, Ri, t‐Ww, L‐Ww	Goetz (2007)
Homopus signatus	ab, ap, am, Lb, Lp, Li, Wwb, Wwi, Ri, L‐dL	Loehr (2004)
Hydromedusa maximiliani	ab, ap, am, Lb, Lp, Li, Wwb, Wwi, Ri, L‐dL	Martins and Souza (2008), Novelli and de Sousa (2008)
Kinosternon flavescens	ab, ap, am, Lb, Li, Wwi, Ri, t‐L, Ww‐WwR	Iverson (1991)
Kinosternon hirtipes	ab, ap, am, Lb, Li, Wwi, Ri, t‐L	Iverson et al. (1991)
Kinosternon scorpioides	ab, am, Lb, Lp, Li, Ww0, Wwi, Ri, t‐L, t‐Le	dos Santos Braga et al. (2021), Iverson (2010)
Kinosternon sonoriense	am, Lb, Lp, Li, Wwb, Wwi, Ri, t‐L	Hensley et al. (2010)
Kinosternon subrubrum	ab, ap, am, Lb, Li, Wwi, Ri, t‐L, L‐Ww	Iverson (1979)
Lepidochelys kempii	ab, ap, am, Lb, Lp, Li, Wwb, Wwp, Wwi, Ri	Spotila (2004)
Lepidochelys olivacea	ab, ap, am, Wwb, Wwp, Wwi, Ri, t‐Ww	Markham and Kirkwood (1988)
Macrochelys temminckii	ab, ap, am, Lb, Lp, Li, Wwp, Wwi, Ri, t‐L	Dobie (1971)
Malaclemys terrapin	ab_T, ap, am, Wwb, Wwi, Ri, t‐Ww_T	Roosenburg and Kelley (1996)
Malacochersus tornieri	ab, ap, am, Lb, Li, L_t, Wwb, Wwi, Ww_t, Ri	Ewert et al. (2004)
Mauremys japonica	ap, am, Lb, Lp, Li, Wwb, Wwi, Ri, t‐L	Yabe (1989)
Mauremys reevesii	ab, am, Wwb, Wwp, Wwi, Ri, t‐Ww	Du et al. (2009)
Mauremys rivulata	ab, ap, am, Lb, Lp, Li, Wwi, Ri, t‐L	Çiçek et al. (2016)
Mauremys sinensis	ap, am, Lb, Lp, Li, Wwb, Wwp, Wwi, Ri, t‐L	Chen and Lue (1998)
Mecistops cataphractus	ab, ap, am, Lp, Li, Ww0, Wwb, Wwi, Ri	
Melanochelys tricarinata	ap, am, Lb, Lp, Li, Wwb, Wwi, Ri, t‐L, L‐Ww	Kumar et al. (2010)
Melanosuchus niger	ab, am, Lp, Li, Wwb, Wwi, Ri, L‐L	Herron (1991)
Myuchelys bellii	ab, ap, am, Lb, Lp, Li, Ww0, Wwi, Ri, t‐L	Fielder et al. (2015)
Natator depressus	ah, ab, ap, am, Lh, Lb, Lp, Li, Ww0, Wwh, Wwp, Wwi, Ri, E0, T‐ah, L0‐Lt, L‐Ww, t‐Ww	Bentley (2017), Limpus (2007), Rusli et al. (2016), Salmon (2017), Stubbs et al. (2019), Venkatesan et al. (2005), Wine (2016)
Osteolaemus tetraspis	ab, ap, am, Lp, Li, Ww0, Wwb, Wwi, Ri	
Paleosuchus palpebrosus	ab, ap, am, Lb, Lp, Li, Wwb, Wwi, Ri, t‐L	Campos et al. (2013)
Paleosuchus trigonatus	ab, ap, am, Lp, Li, Wwb, Wwi, Ri, t‐L, t‐Ww	
Pangshura tecta	ab, ap, am, Lb, Lp, Li, Wwb, Wwi, Ri, t‐L, t‐Ww	Vyas (1979)
Pelodiscus sinensis	am, Lp, Li, Wwb, Wwi, Ri, t‐Ww, T‐ab	Ji et al. (2003)
Pelomedusa subrufa	ab, ap, am, Lb, Lp, Li, Wwb, Wwp, Wwi, L‐N	Strydom (2001)
Pelusios castanoides	ab, ap, am, Lb, Lp, Li, Wwp, Wwi, Ri, t‐L	Gerlach (2008)
Pelusios subniger	ab, ap, am, Lb, Lp, Li, Wwp, Wwi, Ri, t‐L	Gerlach (2008)
Platysternon megacephalum	ab, ap, am, Lp, Li, Wwb, Ri, L‐Ww	Sung et al. (2014), Sung et al. (2015)
Podocnemis expansa	ab, ap, am, Lb, Lp, Li, Wwb, Wwp, Wwi, Ri, t‐L_e, t‐L	Chinsamya and Valenzuela (2008), Magalhāes et al. (2017)
Podocnemis lewyana	ap, am, Lb, Lp, Li, Wwb, Wwi, Ri, L‐dL, T‐ab	Páaez et al. (2015), Páez et al. (2009)
Podocnemis unifilis	ab, ap, am, Lb, Lp, Li, Wwb, Wwp, Wwi, Ri, t‐L_f, t‐Ww_f	Meers et al. (2016), Miorando et al. (2015)
Psammobates geometricus	ab, ap, am, Lb, Lp, Li, Wwi, Ri, L‐dL	Baard (1995)
Psammobates oculiferus	am, Lb, Lp, Li, Wwp, Ri, t‐L, t‐Ww	Keswick (2012)
Pseudemydura umbrina	ab, ap, am, Lb, Lp, Li, Wwb, Wwp, Wwi, Ri, L‐Ww, t‐L_f, t‐Ww_f, T‐JO	Arnall (2018), Arnall et al. (2015), Burbidge (1981), Burbidge et al. (2010)
Pseudemys alabamensis	ab, ap, am, Lb, Li, Ri, t‐L, L‐Ww	Graham (1971)
Pseudemys concinna	ab, ap, am, Lb, Li, Wwb, Wwi, Ri, t‐L	Dreslik (1997)
Pseudemys nelsoni	ab, ap, am, Lb, Li, Wwb, Wwi, Ri, L0‐Lt	Munscher et al. (2015)
Pseudemys peninsularis	ap, am, Lb, Li, Wwi, Ri, L0‐Lt	Munscher et al. (2015)
Pseudemys texana	ab, ap, am, Lb, Lp, Li, Wwi, Ri, t‐L	Lindeman (2007)
Rhinemys rufipes	ab, ap, am, Lb, Lp, Li, Wwp, Wwi, Ri, L0‐Lt	Magnusson et al. (1997)
Sternotherus depressus	ab, ap, am, Lb, Li, Wwi, Ri, L‐r	Melancon et al. (2011)
Sternotherus minor	ab, ap, am, Lb, Lp, Li, Wwb, Wwi, Ri, L‐r	Becker (2003), Cox et al. (1991)
Sternotherus odoratus	ab, ap, am, Lb, Li, Wwi, Ri, t‐L	Ernst (1986)
Stigmochelys pardalis	ab, ap, am, Li, Wwb, Wwi, Ri, t‐Ww, L‐Ww	Ritz, Hammer, et al. (2010)
Terrapene carolina	ab, ap, am, Lb, Lp, Li, Wwi, Ri, t‐L	Ernst et al. (1998)
Terrapene ornata	ab, ap, am, Lb, Li, Wwi, Ri, t‐L, L‐Ww	Skorczewski and Andersen (2021)
Testudo graeca	ab_T, ap, am, Wwb, Ri, t‐Ww	Hichami et al. (2016), Ritz et al. (2012)
Testudo hermanni	ab, ap, am, Lp, Li, Wwb, Wwi, Ri, t‐Ww	Ritz et al. (2012)
Tomistoma schlegelii	ab, ap, am, Lp, Li, Ww0, Wwi, Ri	
Trachemys scripta	ab, ap, am, Lb, Li, Wwb, Wwi, Ri, t‐L	Frazer et al. (1990)
Trionyx triunguis	am, Lp, Li, Ww0, Wwb, Wwi, Ri, t‐Wwe, t‐Wde, t‐JOe	Leshem et al. (1991)

**TABLE A2:** The codes of the data types as presented in Table 1. Zero variate data left, uni‐variate data right. Life history events: 0 start development, h hatch, b birth, p puberty, m death, i death. T stands for temperature

Code	Description	Code	Description
ah	age at h	t‐Le	time, embryo length
ab	age at birth	t‐L	time, length
ab_T	age at birth (several T)	t‐L_T	time, length (several T)
ap	age (or time since birth) at p	t‐L_f	time, length (several f)
am	age at death (life span)	t‐L_fT	time, length (several f, T)
Lh	length at h	t‐Wwe	time, embryo wet weight
Lb	length at b	t‐WwYe	time, embryo yolk wet weight
Lp	length at p	t‐WwVe	time, embryo wet weight excluding yolk
Li	length at i	t‐Ww	time, wet weight
L_t	length at time t	t‐Ww_f	time, wet weight (several f)
Ww0	wet weight at 0	t‐Ww_T	time, wet weight (several T)
Wwh	wet weight at h	t‐Wde	time, embryo dry weight (total)
Wwb	wet weight at b	t‐JOe	time, embryo O_2_ consumption
Wwp	wet weight at p	t‐JOe_T	time, embryo O_2_ cons (several T)
Wwi	wet weight at i	L‐L	length, length (different length measures)
Ww_L	wet weight at length	L‐dL	length, change in length
Ww_t	wet weight at time	L0‐Lt	length at capture, length at recapture
E0	reserve energy at 0	L‐Ww	length, wet weight
Ri	reproduction rate at i	L‐r	length, specific growth rate
R_L	reproduction rate at length	L‐N	length, number of eggs/offspring
pAi	maximum assimilation rate (energy)	Ww‐WwR	wet weight, clutch wet weight
JXi	food consumption at i	T‐ah	temperature, age at h
		T‐ab	temperature, age at b
		T‐JO	temperature, O_2_ consumption
